# Massive neonatal intracranial hemorrhage caused by bromadiolone: A case report

**DOI:** 10.1097/MD.0000000000008506

**Published:** 2017-11-10

**Authors:** Mingsheng Ma, Mengqi Zhang, Xiaoyan Tang, Zhenghong Li

**Affiliations:** Department of Pediatrics, Peking Union Medical College Hospital, Chinese Academy of Medical Sciences and Peking Union Medical College, Beijing, China.

**Keywords:** clinical toxicology, intracranial hemorrhage, neonatal, superwarfarin

## Abstract

**Rationale:**

Bromadiolone, often called a super-warfarin, is a potent rodenticide with long half-life. Skin and mucosal bleeding is the most common clinical manifestations of its intoxication. Bromadiolone intoxications in adults and children have been reported, but this phenomenon is rarely seen in fetuses. This paper presents a case of neonate with massive intracranial hemorrhage mediated by bromadiolone intoxication, highlighting that the bromadiolone is potentially lethal to the fetus.

**Patient Concerns:**

The male neonate presented with poor respiratory effort, decreased muscle tone, and pallor at birth. He developed generalized seizures on day 1 of life. His mother suffered from bleeding of oral mucosa and the subsequent lab screening for toxicants showed a bromadiolone level of 126 ng/mL.

**Diagnoses:**

Laboratory tests revealed that prolonged prothrombin time (PT) and activated partial thromboplastin time (aPTT). A computed tomography (CT) of his head revealed a severe subdural hematoma, which lead to midline shift, bilateral intraventricular hemorrhage, and subarachnoid hemorrhage. Serum from cord blood was collected and screened for toxicants. The result returned with a bromadiolone level of 94 ng/mL.

**Interventions:**

The neonate was treated with vitamin K, fresh-frozen plasma, and red blood cells.

**Outcomes:**

His parents required termination of all treatments, and the neonate unfortunately died shortly after.

**Lessons:**

Through clinical experience from this case, we believe that bromadiolone can be passed down to the fetus via placenta. Neonatal intracranial hemorrhage caused by bromadiolone is rare but potentially lethal. Pregnant women should be informed of the serious side effects of bromadiolone and this poisonous reagent should be avoided in any period during pregnancy.

## 1 Introduction

Bromadiolone, often called a superwarfarin, is a second-generation 4-hydroxycoumarin derivative and a long-acting anticoagulant. As one of the most potent rodenticides, bromadiolone is commonly used worldwide. The most common route of exposure to bromadiolone is ingestion, but unknown history of toxicant intake may impede the physician from making a correct diagnosis. The most typical clinical feature of superwarfarin intoxication is bleeding, which can occur on any mucosal site or organ.^[1]^

Intracranial hemorrhage of the full term infant may be associated with bleeding disorder (such as alloimmune thrombocytopenia, vitamin K deficiency bleeding, hemophilia, and other coagulation abnormalities), trauma, rupture of a vascular malformation, and sinovenous thrombosis.^[2]^ Bromadiolone intoxication in adults and children have been reported in China, with relatively mild clinical features, but this problem is rarely seen in fetuses.^[3]^ Recognition of the severe coagulopathy in newborn resulting from bromadiolone poisoning is severely lacking. We report a neonatal case involving bromadiolone.

## 2 Ethical statement

The study mainly involves retrospective observations of a patient; therefore, ethical approval was not needed. Informed consent was obtained from the patient's father for publication.

### 2.1 CASE

A boy was delivered at 38 weeks of gestation to a 25-year-old gravida1 paral1 by emergency cesarean section soon after the coagulation function of his mother had become normal. His mother had bled from oral mucosa for 3 days because of an unknown reason. Blood tests showed a prolonged prothrombin time (PT) exceeding 70 s (normal: 10.4–12.6 s) and activated partial thromboplastin time (aPTT) of 110.5 s (normal: 74.0–110.0 s). The addition of normal plasma in a 1:1 ratio resulted in complete correction of the coagulation abnormalities, and a coagulation factor deficiency was suspected. The results of factor assay were associated with vitamin K deficiency: the levels of factor II, factor VII, factor IV, and factor X were sharply low, but factor V and factor VIII were within normal ranges. The mother was given vitamin K intravenously, and the oral bleeding stopped after 8 hours. Serum screening for toxicant by using gas chromatography/mass spectrometry (GC/MS) was taken 6 hours after admission and the result showed a bromadiolone level of 126 ng/mL.^[4]^ She denied intentional ingestion of any kind of rodenticides. The most likely route of exposure might be ingestion of the food contaminated by bromadiolone.

The boy's APGAR scores^[5]^ were 2 (Pulse: 2), 5 (Activity: 1;Pulse: 2;Grimace: 1;and Respiratory effort: 1), and 5 (Activity: 1;Pulse: 2;Grimace: 1;Respiratory effort: 1) at 1, 5, and 10 minutes respectively. Blood gas analysis showed pH of 7.249, *P*_CO2_of 55.9mmHg, *P*_O2_of 32mmHg, and BE of −4.2mmol/L. Immediate intubation and resuscitation was necessary. The boy showed poor respiratory effort, decreased muscle tone, and pallor. He was transferred to the neonatal intensive care unit for further treatment. The patient presented no history of trauma and bleeding diathesis.

Upon admission, the boy's measurements were as follows: weight 3120 g; length 48 cm; and fronto-occipital head circumference 37 cm. All of these measurements were appropriate for the boy's gestational age. The boy presented with bulging anterior fontanelle, a heart rate of 169 bpm, and blood pressure of 68/40 mmHg. No external signs of spontaneous hemorrhage were noted. The pupils were dilated and fixed to light. Corneal, Moro, and rooting reflexes were absent. Hepatomegaly and splenomegaly were not found.

Laboratory tests revealed hemoglobin level of 74 g/L, white blood cell count of 17.57 × 10^9^/L, and platelet count of 264 × 10^9^/L. PT exceeded 70 s (upper limit of reference), and aPTT was greater than 150 s (upper limit of reference). Fibrinogen (3.02 g/L) and thrombin time (16.4 s) were normal. Serology assay showed the following values: ALT: 5U/L; Alb: 28 g/L; TBil: 27.4 μmol/L; Cr: 57 μmol/L; CK: 353U/L; and CKMB: 6.3 μg/L.

**Figure 1:**
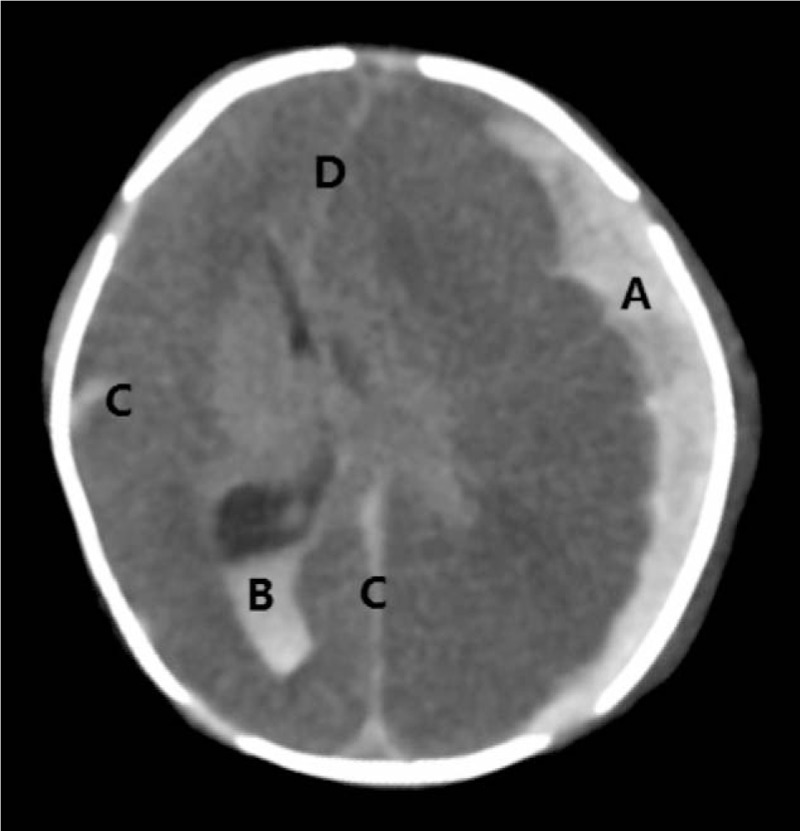
The head CT scan. It was shown that a severe subdural hematoma (A) causing midline shift (D), bilateral intraventricular hemorrhage (B), and subarachnoid hemorrhage (C).

The neonate was treated with vitamin K, fresh-frozen plasma, and red blood cells. His oral mucosa started to bleed at 2 hours after birth. Muscle tone increased at 4 hours after birth. Generalized seizures started on day 1 of life. A head computed tomography showed a severe subdural hematoma causing midline shift, bilateral intraventricular hemorrhage, and subarachnoid hemorrhage (Fig. 1). Serum from cord blood was screened for toxicants. The result showed bromadiolone level of 94 ng/mL. His parents requested to stop all treatment, and the neonate died shortly after. Autopsy was not obtained from the parents.

## 3 Discussion

Bromadiolone, called a superwarfarin, is a potent anticoagulant rodenticide. Its long elimination half-life leads to a long-lasting pharmacodynamic effect. Its effect appears after a latency period of several days after ingestion. Bromadiolone obeys the elimination kinetics of a 2-compartment model, with a rapid, fairly steep decline phase (half-life of 3.5 days), followed by a slower termination phase (half-life of 24 days).^[6]^ It is mainly excreted as metabolites in the urine and feces.^[7]^

Parallel to the growing distribution of bromadiolone for rodent control, the number of human-poisoning instances is increasing. The most common clinical feature of superwarfarin intoxication is bleeding, which can occur from any mucosal site or organ. Reports on neonates with superwarfarin poisoning are rare. Mehlhaff et al^[8]^ described one case of neonatal superwarfarin (brodifacoum) poisoning. They observed severe intracranial hemorrhage, which was similar to the findings in our case. Jie et al^[9]^ reported that a neonate was delivered stillborn at 37 weeks, and autopsy showed severe hemorrhage in the brain because his mother accidentally ingested rodenticide (brodifacoum). By contrast, Zurawski et al^[10]^ described another case of superwarfarin poisoning during pregnancy without fetal hemorrhage. The authors speculated that the brodifacoum molecule might not cross the placenta. The bromadiolone level of cord blood in our case was 94 ng/mL. This result demonstrates that long-acting anticoagulants actually cross the placenta and cause fetal coagulopathy, and the severity of symptoms is closely related to bromadiolone level.

Most of the adult patients with superwarfarin intoxication recovered without sequelae.^[11]^ However, the fetal cases presented serious intracranial hemorrhage and were even fatal.^[9]^ Intracranial hemorrhage from a bleeding disorder is rare in the full term newborn but tends to be more severe and devastating.^[12]^ Similar to our case, seizure was seen in most infants with intracranial hemorrhage. Intracranial hemorrhage in the full term newborn involving multiple compartments, namely, subdural, subarachnoid, intraventricular, or intraparenchymal, were included. Our case demonstrated subdural, intraventricular, and subarachnoid hemorrhage through head computed tomography.

In our case, bleeding in-utero was observed because the newborn was anemic at birth. Although his mother's coagulation function normalized after treatment with vitamin K and fresh-frozen plasma, the coagulation function of the neonate was still not working normally. Fetal coagulopathy may not be resolved until weeks after maternal ingestion of bromadiolone. For expectant mothers with coagulation disorders, the optimal mode of delivery remains controversial.^[6]^ Apparently, vaginal delivery increases the risk of hemorrhages (intra- and extracranial) compared with cesarean delivery. Therefore, we propose that cesarean delivery soon after correction of the maternal coagulopathy is the best approach. Currently, no consensus in the guideline for the treatment of human bromadiolone poisoning has been achieved.^[13]^To the best of our knowledge, vitamin K and transfusion with fresh-frozen plasma may be important treatments for newborn with coagulopathy originating from superwarfarin toxicity.

The limitation of our work is that we can’t make sure the clearly cause of death. In this case, autopsy was refused, so we could only speculate that he died from severe intracranial hemorrhage and hemorrhage of other organs. Histopathology could provide more information on tissue distribution of bromadiolone. Necropsy of animal poisoned by superwarfarin revealed multifocal hemorrhage mainly in pericardial sac, muscles, subcutaneous tissue, and the brain.^[14]^

In conclusion, on the basis of the clinical experience from this case, we suggest that bromadiolone can be passed to the fetus through placenta and that this poison should be avoided in any period during pregnancy. Neonatal intracranial hemorrhage caused by bromadiolone is rare but critical and potentially lethal. Pregnant women should be informed of the serious effects of bromadiolone to fetus.
